# Farewell and thanks for all the fish

**DOI:** 10.1128/msystems.00671-25

**Published:** 2025-05-29

**Authors:** Jack A. Gilbert

**Affiliations:** 1 University of California San Diego 8784, La Jolla, California, USA

## EDITORIAL

As I prepare to step down from my role as editor in chief of *mSystems* at the end of June 2025, I find myself reflecting on the extraordinary journey we have shared since the journal’s inception. It has been an immense privilege to serve as the inaugural editor in chief, a role I undertook with enthusiasm, optimism, and, admittedly, a touch of trepidation. Ten years later, my enthusiasm and optimism remain undimmed, while the trepidation has been replaced by a profound sense of gratitude and humility.

First and foremost, I wish to extend my heartfelt thanks to the American Society for Microbiology (ASM) and the ASM Journals Board for placing their trust in me to lead this remarkable experiment back in 2015. The decision to launch *mSystems* was bold and visionary, aimed at providing a dynamic platform that would support the dissemination of research at the scientific boundaries of microbial systems biology. From the outset, our mission was ambitious: to foster rigorous interdisciplinary dialog and to explore frontiers that transcend traditional disciplinary constraints. Obviously, this led to a lot of questions about what type of research *mSystems* would publish, but I think we have proven over a decade that we publish work that focuses on microbial cell and ecosystem biology, as it relates to how the components of these systems connect with each other. Admittedly, this remains a broad remit—but so is systems biology. I have often said that if you build a platform right, it will evolve to support the needs of the community.

The success of this mission owes immeasurably to the exceptional staff at ASM Journals, whose tireless support, professionalism, and ingenuity have been fundamental to our achievements. Without their dedication, the journal simply would not have reached the high standards of scientific publishing and community engagement that we have come to expect and cherish.

My deepest appreciation also goes to the incredible community of scientists who have served as editors, senior editors, reviewers, and consultants for *mSystems*. Peer review is an imperfect yet absolutely essential mechanism for upholding scientific integrity and ensuring rigorous inquiry. Over the past decade, I have been humbled by the collective effort, commitment, and goodwill of colleagues around the world who generously contributed their time and expertise to strengthen the quality of our published work.

As I prepare to pass the baton to my successor, Professor Ashley Shade, I do so with tremendous pride and confidence. I have had the joy of witnessing Ashley’s career flourish from her early days in graduate school to her present position as a leading professor in microbial ecology. Ashley embodies the energy, enthusiasm for innovation, and meticulous attention to detail that *mSystems* requires as it continues to evolve. Her leadership will undoubtedly enhance the journal’s legacy of interdisciplinary rigor and integrity, critical for maintaining public trust in science and the scientific method.

In leading this journal, I quickly discovered the necessary humility that comes with being a steward of scientific communication. Editors wield considerable influence as gatekeepers, yet this power must always be exercised with transparency and an openness to the complexities inherent in every submission. Throughout my tenure, I have continued to learn and, by doing so, have understood that no individual can fully grasp every nuance of the diverse fields encompassed by microbial systems biology. The strength of *mSystems* has always derived from the diversity of its community-focused spirit, where collective insight enriches individual understanding.

Indeed, it is this social ethos that I consider our greatest accomplishment. *mSystems* is truly a community journal, built by scientists for scientists, providing an inclusive, rigorous, and innovative platform for the advancement of knowledge. Our flexible and interdisciplinary nature has allowed us to challenge traditional barriers, champion transparent data sharing, and cultivate collaborations that span microbiology, chemistry, engineering, medicine, computational biology, social sciences, and beyond. Our diversity is our strength.

As I step away, my hope for *mSystems*—and for the community it supports—is that it continues to flourish as a beacon of innovation, integrity, and inclusivity. Thank you again to ASM, to our staff, our editorial and reviewer community, our dedicated readers, and all our contributing authors. Your collective passion, curiosity, and dedication to excellence have profoundly enriched my experience and shaped this journal’s identity and impact.

With profound gratitude and optimism for the future.

